# Comparison of presentation, treatment and follow-up outcomes in first and second wave cohorts of multisystem inflammatory syndrome in children

**DOI:** 10.3389/fped.2026.1710510

**Published:** 2026-01-29

**Authors:** Münevver Yılmaz, Dolunay Gürses, Merve Oğuz, Gülşah Kılbaş, Selçuk Yüksel

**Affiliations:** 1 Department of Pediatric Cardiology, Faculty of Medicine, Pamukkale University, Denizli, Türkiye; 2 Department of Pediatric Rheumatology, Faculty of Medicine, Trakya University, Edirne, Türkiye; 3 Department of Pediatric Rheumatology, Faculty of Medicine, Çanakkale Onsekiz Mart University, Çanakkale, Türkiye

**Keywords:** 2019 coronavirus disease, echocradiography, multisystem inflammatory syndrome in children, SARS-CoV-2, time series data

## Abstract

**Objective:**

Multisystem inflammatory syndrome (MIS-C) is life-threatening complication of coronavirus disease 2019 (COVID-19) in children. The objective of this study is to compare the clinical features, and follow-up results of MIS-C patients between the first and second waves of the disease.

**Methods:**

This study was conducted retrospectively in children with MIS-C who were hospitalized in Pamukkale University Hospital. The first wave was defined as October 2020-June 2021 and the second wave as August 2021-February 2022. The clinical characteristics of the patients were recorded. The patients were evaluated by echocardiography at the sixth and twelfth months.

**Results:**

Seventy were diagnosed in the first wave and 32 were diagnosed in the second wave, with 102 children included in the study. Pulmonary system involvement was more common in the first wave (*p* = 0.043). In the second wave, IL-6 (*p* = 0.033), ESR (*p* = 0.001), ALT (*p* = 0.048), LDH (*p* = 0.009), lipase (*p* = 0.05), and D-Dimer (*p* = 0.027) were found higher. Elevated ESR (*p* < 0.001), LDH (*p* = 0.038), and ALT (*p* = 0.008), thrombocytopenia (*p* = 0.011), and pericardial effusion were more frequent in the second group (*p* = 0.024). At the 12th month evaluation, it was observed that coronary aneurysm persisted in one patient each in the first and second waves groups.

**Conclusions:**

The findings of this study revealed a significant increase in laboratory parameters of the MIS-C patients in time throughout the COVID-19 waves. There was no significant difference between the cohorts about different waves in terms of clinical findings and treatments. The echocardiographic findings of the patients have also not differed significantly between follow-ups.

## Introduction

Multisystem Inflammatory Syndrome in Children (MIS-C) is a newly identified serious hyperinflammatory disease linked to the severe acute respiratory syndrome coronavirus 2 (SARS-CoV-2) (1–3). In addition to mucocutaneous involvement, as in Kawasaki disease, cardiovascular, gastrointestinal, hematological, neurological, pulmonary, and renal involvements are also observed. The coronavirus disease 2019 (COVID-19) tends to be mild or asymptomatic in most children. However, children affected by MIS-C require hospitalization, and some of these children, who are in critical condition, are admitted to intensive care units (ICUs) (1–5).

Since the declaration of COVID-19 as a pandemic, there have been several waves in the course of the infection worldwide (6, 7). In line with these fluctuations in the circulation of the SARS-CoV-2 virus, there have also been fluctuations in the number of MIS-C patients (1, 4, 5). However, the comparative data on the clinical features and outcomes in patients with MIS-C between different waves are insufficient. There are only a few studies in the literature that compared the first wave of patients with patients of other waves. Then again, the findings of these studies are contradictory in that some reported a decrease in disease severity over time, whereas others reported an increase, and some reported no difference in clinical features (1, 4–6, 8). On the other hand, there is no study in the literature that compared the relevant results in the medium-long term.

Understanding the evolution of MIS-C over time may help to understand the pathophysiology and clinical presentation of this newly identified disease. In this context, the objective of this study is to compare the clinical features and the 6-month and 12-month follow-up results of the MIS-C patients followed up in the hospital where this study was conducted since the beginning of the COVID-19 pandemic between the first and second waves of the disease.

## Methods

This study was carried out retrospectively in children with MIS-C who were hospitalized in the Pediatric Cardiology Service of Pamukkale University Medical Faculty Hospital between October 2020 and February 2022. The diagnosis of MIS-C was made based on the criteria of the United States Centers for Disease Control and Prevention (CDC) and the World Health Organization (WHO) (9, 10). Patients who were diagnosed and started to receive treatment at another healthcare facility before they were referred to the hospital where this study was conducted were excluded from the study.

Demographic characteristics, admission complaints, and physical examination findings of the patients were recorded. Additionally, laboratory parameters levels at the time of admission and discharge as well as the maximum or minimum values of these parameters reached during the follow-up were also recorded. The reference levels for BNP and D-dimer were set at 1,000 pg/mL and 550 ng/mL, respectively. The reference levels for other laboratory parameters were determined by the age of the patients in the study group (1, 11).

All patients' electrocardiograms (ECG) were taken and echocardiographic assessment was performed. Pathological findings in the echocardiographic evaluation included left ventricular dysfunction, valve regurgitations, coronary artery lesions, pericarditis/pericardial effusion. Left ventricular systolic dysfunction was defined as ejection fraction less than 55% (1, 4, 11). The treatment modalities patients received, vasoactive-inotropic score, and their length of hospital stay (LoS) were recorded (12). The patients were followed up in the pediatric cardiology outpatient clinic after discharge and were evaluated by echocardiography during the 6-month and 12-month follow-up visits.

**Figure 1:**
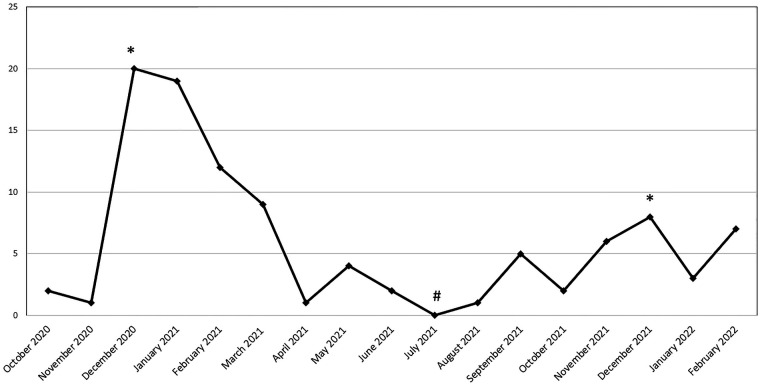
Distribution of the number of MIS-C patients by months.

The curve of MIS-C diagnosis dates was reviewed to assess the outcomes of cases over time. Two peak dates (*) where the number of patients was the highest, and one peak date (#) where the number of patients was the lowest were determined in the graph (Figure 1). MIS-C patients diagnosed during the pandemic were evaluated in two periods, i.e., October 2020–June 2021 and August 2021-February 2022.

Prior to the initiation of the study, approval was obtained from the Pamukkale University Faculty of Medicine non-invasive clinical research ethics committee (27.09.2022/14).

SPSS 18.0 [Predictive Analytics SoftWare (PASW) Statistics for Windows, version 18.0, SPSS Inc., Chicago, IL, U.S., 2009] software package was used for the statistical evaluation of the data. The conformance of the data to the normal distribution was tested by the Kolmogorov–Smirnov test. Student's *t*-test and Mann–Whitney *U*-test were used to evaluate the parametric and non-parametric data, respectively. Pearson's chi-squared and Fisher's exact tests were used to evaluate the categorical data. Results were expressed as numbers and percentage values in the case of categorical variables, mean ± standard (min-max) values in the case of parametric data, and median values (IQR) in the case of non-parametric data. Multivariate logistic and linear regression analyses were performed to account for potential confounding variables, including age and gender. Probability (*p*) values of <0.05 were deemed to indicate statistical significance.

## Results

A total of 102 patients who were followed up with the diagnosis of MIS-C were included in the study. Seventy and 32 patients were diagnosed during the first-wave (October 2020–June 2021) and second-wave (August 2021-February 2022), respectively. Two patients in the second wave group, who were diagnosed and started treatment at another hospital, were excluded from the study. The mean age of the patients was 8.11 ± 4.5 (min.1-max.17) years. The male/female ratios of the study sample and the first and second-wave cohorts were calculated as 57/45, 41/29, and 16/16, respectively (*p* = 0.419) (Table 1).

**Table 1:** Demographic and clinical findings of the patients.

Variables		Study group (*n*: 102)	First wave cohort (*n*: 70)	Second wave cohort (*n*: 32)	*p*-value
Gender (M/F)		57/45	41/29	16/16	0.419
Age, years	mean ± SD (min-max)	8.1 ± 4.5 (1–17)	8.1 ± 4.6 (1–17)	8.1 ± 4.4 (2–17)	0.983*
0–6 years		36 (%35.3)	23 (%32.9)	13 (%40.6)	0.5646
6–12 years		44 (%43.1)	30 (%42.9)	14 (%43.8)	
12–18 years		22 (%21.6)	17 (%24.2)	5 (%15.6)	
Height, cm	mean ± SD (min-max)	130.2 ± 27.1 (75–186)	130.1 ± 27.6 (75–186)	130.3 ± 26.4 (88–178)	0.968*
Body weight, kg	median (IQR)	26.5 (18–49)	26 (18–49.3)	28.5 (18–46.3)	0.92**
Obesity		14 (%13.7)	11 (%15.7)	3 (%9.4)	0.540**
Body temperature, °C	mean ± S (min-max)	39.1 ± 0.7 (38 -40.5)	39 ± 0.7 (38–40.5)	39.3 ± 0.6 (38.2 -40.5)	0.238*
Duration of fever, days	Median (IQR)	4 (3–5)	4 (2–5)	4 (3–6)	0.133**
Changes in oral mucosa		32 (%31.4)	21 (%30)	11 (%34.3)	0.659
Rashes		48 (%47.1)	33 (%47.1)	15 (%46.9)	0.98
Maculopapular		37 (%77.1)	27 (%81.8)	10 (%66.6)	0.086
Urticarial		5 (%10.4)	1 (%3)	4 (%26.7)	
Erythematous		5 (%10.4)	4 (%12.1)	1 (%6.7)	
Stevens-Johnson syndrome		1 (%2.1)	1 (%3)	0 (%0)	
Site of rashes					
Trunk		13 (%27.1)	5 (%15.2)	8 (%53.4)	**0**.**025**
Extremity		12 (%25)	10 (%30.3)	2 (%13.3)	
Whole body		23 (%47.9)	18 (%54.5)	5 (%33.3)	
Changes in extremities		5 (%4.9)	2 (%2.9)	3 (%9.4)	0.176
Conjunctivitis		51 (%50)	34 (%48.6)	17 (%53.1)	0.67
Stomach ache		61 (%59.8)	44 (%62.9)	17 (%53.1)	0.352
Vomiting		49 (%48)	34 (%48.6)	15 (%46.7)	0.874
Diarrhea		35 (%34.3)	27 (%38.6)	8 (%25)	0.18
Irritability		33 (%32.4)	21 (%30)	12 (%37.5)	0.452
Headache		22 (%21.6)	15 (%21.4)	7 (%21.9)	0.806
Change in consciousness		4 (%3.9)	2 (%2.9)	2 (%6.2)	
Stroke		1 (%1)	1 (%1.4)	0 (%0)	
Seizure		1 (%1)	1 (%1.4)	0 (%0)	
Meningismus		5 (%4.9)	3 (%2.9)	2 (%6.2)	
Respiratory distress		6 (%5.9)	5 (%10)	1 (%3.1)	0.221
Cough		13 (%12.7)	11 (%15.9)	2 (%6.2)	
Hypotension or shock		32 (%31.4)	23 (%32.9)	9 (%28.1)	0.633
Chest pain		9 (%8.8)	6 (%8.6)	3 (%9.4)	0.580
Cervical lymphadenopathy		9 (%8.8)	4 (%5.7)	5 (%15.6)	0.135
Arthritis		11 (%10.8)	7 (%10)	4 (%12.5)	0.737
Systemic involvement					
Gastrointestinal		88 (%86.3)	59 (%84.3)	29 (%90.6)	0.54
Hematological		82 (%80.4)	56 (%80)	26 (%81.2)	0.883
Cardiac		76 (%74.5)	50 (%71.4)	26 (%81.3)	0.291
Mucocutaneous		68 (%66.7)	47 (%67.1)	21 (%65.6)	0.88
Neurological		33 (%32.4)	22 (%31.4)	11 (%34.4)	0.768
Pulmonary		22 (%11.6)	19 (%27.1)	3 (%9.4)	**0**.**043**
Renal		15 (%14.7)	12 (%17.1)	3 (%9.4)	0.379

The *p*-values in bold are statistically significant.

* *p*-value was obtained from the Mann–Whitney *U*-test.

** *p*-value was obtained from the student's *t*-test.

There was an underlying disease in 9 (12.9%) patients diagnosed during the first-wave and 3 (9.4%) patients diagnosed during the second-wave (*p* = 0.749). There was also no significant difference between the first and second-wave cohorts in terms of obesity (*p* = 0.54) (Table 1).

The most common clinical finding was fever, followed by abdominal pain, conjunctivitis, vomiting, rash, and diarrhea (Table 1). There was no significant difference between the first and second-wave cohorts in the frequency of clinical findings. The only significant difference between the first and second-wave cohorts was that rashes were more frequently observed in the whole body in the first-wave cohort and the trunk in the second-wave cohorts (*p* = 0.025) (Table 1). Cough was present in 11 (15.7%) patients in the first-wave cohort and only in one (3.1%) patient in the second-wave cohort; however, the difference was not significant (*p* = 0.121) (Table 1). None of the patients had been vaccinated against SARS-CoV-2.

In terms of system involvements, the most common one was gastrointestinal system involvement, followed by hematological, cardiac, mucocutaneous, neurological, pulmonary, and renal system involvements, respectively (Table 1). Among these system involvements, only pulmonary system involvement was more common in the first-wave cohort (*p* = 0.043).

The laboratory findings of the patients are given in Table 2 and Table 3. The baseline and peak values of C-reactive protein (CRP), procalcitonin (PCT), interleukin-6 (IL-6), and ferritin were higher in the second-wave cohort (Table 2). In addition, frequency of elevated IL-6 and ferritin values were significantly higher in the second-wave cohort, both at admission and as of the peak value (Table 3). There was significant difference between the cohorts only in terms of both admission and peak IL-6 levels (*p* = 0.033 and *p* = 0.025, respectively), (Table 2). The erythrocyte sedimentation rate (ESR) values measured at admission and the frequency of elevated ESR values were significantly higher in the second-wave cohort than in the first-wave cohort (*p* = 0.001 and *p* < 0.001, respectively). Thrombocytopenia was more common in the second-wave cohort both at admission and during the follow-up period (*p* = 0.011 and *p* = 0.025, respectively) (Table 3). In addition, platelet values were lower, albeit not significantly, in the second-wave cohort. The frequency of elevated lactate dehydrogenase (LDH) levels at admission and the peak LDH levels were significantly higher in the second-wave cohort (*p* = 0.038 and *p* = 0.009, respectively). The frequency of elevated transaminase levels and alanine transaminase (ALT) values were significantly higher in the second-wave cohort (*p* = 0.008 and *p* = 0.048, respectively). The peak lipase level was borderline high in the second-wave cohort (*p* = 0.05). Similarly, peak D-Dimer values were found to be higher in the second-wave cohort (*p* = 0.027) (Table 2). After adjusting for age and sex, elevated ESR values at admission continued to be significantly more frequent in the second wave (adjusted OR = 4.9, *p* = 0.001). Similarly, ESR levels at admission were independently higher in the second wave cohort (β = 14.5 mm/s, *p* = 0.001). Thrombocytopenia at admission also remained significantly associated with the second wave after adjustment.

**Table 2:** Laboratory findings of the patients.

Variables		Study group (*n*: 102)	First wave cohort (*n*: 70)	Second wave cohort (*n*: 32)	*p*-value
CRP (mg/dL)	adm	144.7 ± 83.1 (8.2–339)	140.6 ± 81.7 (8.2–339)	153.5 ± 86.6 (14.5–330)	0.469*
	max	165.3 ± 85.1 (87–374)	160.6 ± 79.8 (8.7–339)	175.5 ± 96.2 (14.5–374)	0.415*
	d/c	2.8 (1.05–6.25)	3.01 (1.28–7.62)	2.51 (0.51–5.15)	0.096
PCT (mg/dL)	adm	1.94 (0.6–6)	1.53 (0.5–5.33)	2.82 (1.03–7.45)	0.156
	max	2.52 (0.74–11.95)	2.01 (0.64–10.14)	3.3 (1.03–20)	0.360
	d/c	0.08 (0.05–0.17)	0.09 (0.05–0.17)	0.08 (0.04–0.17)	0.760
ESR (mm/s)	adm	35.2 ± 20.8 (2–94)	30.8 ± 19 (2–85)	44.8 ± 21.6 (6–94)	**0**.001*
	d/c	18.5 (12–31.25)	18 (12–29)	23 (15.25–31.75)	0.356
IL-6 (pg/mL)^a^	adm	83.2 (11.2–192)	47.1 (8.97–164)	125 (34.18–324.25)	**0**.**033**
	max	93 (19.95–197.5)	52.1 (10.58–178)	148 (39.95–367.5)	**0**.**025**
	d/c	1.5 (1.5–2.99)	1.51 (1.5–3.68)	1.5 (1.5–2.91)	0.438)
Ferritin (µg/L)	adm	296.5 (167–539.8)	255 (146–520.3)	374 (201.8–593.5)	0.085
	max	357.5 (210–637)	324 (207.3–605)	454.5 (272–1,118.5)	0.054
	d/c	154 (76.7–235)	159 (88.15–233.5)	83.3 (66–241)	0.095
Hemoglobin (g/dL)	adm	11.2 ± 5.7 (1.72–16.3)	12.18 ± 1.63 (8.7–16.3)	11.61 ± 1.47 (8.4–14.4)	0.096*
	d/c	11.88 ± 1.83 (7.3–16.5)	11.9 ± 1.5 (7.3–16.5)	11.84 ± 2.44 (7.8–14.42)	0.405*
Leukocyte (10³/µL)	adm	9.65 (6.9–15.1)	10.82 (6.96–15.6)	8.58 (6.37–13.4)	0.105
	d/c	14.2 ± 6.1 (1.31–30.36)	14.8 ± 6.4 (1.31–30.36)	12.8 ± 5.2 (4.16–27.1)	0.125*
Lymphocyte (10³/µL)	adm	1.15 (0.8–2.03)	1.15 (0.81–2.05)	1.2 (0.75–2.01)	0.84
	min	0.9 (0.61–1.46)	0.92 (0.61–1.48)	0.85 (0.58–1.47)	0.974
	d/c	4.01 (2.42–6)	4.01 (2.38–6.6)	3.91 (2.44–5.56)	0.727
Platelet (10³/µL)	adm	211.5 (149.5–269.2)	219.5 (156.75–270.75)	177.5 (118.5–246.5)	0.131
	min	174 (123.25–243.75)	184.5 (139.75–246)	153 (105.5–239.5)	0.192
	d/c	451 (319–591)	459.5 (346.75–599.75)	402 (294.5–537)	0.185
Troponin (ng/L)	adm	7 (3.83–20.33)	7.37 (3.83–20.03)	6.74 (3.4–20.93)	0.726
	max	14 (5.68–32.25)	15.15 (5.25–36.28)	11.55 (5.86–28.7)	0.812
	d/c	3.64 (3–6.03)	3.83 (3–6.3)	3 (3–4.87)	0.076
BNP (pg/mL)	adm	710 (186.5–3,524.25)	488 (130.45–3,093.75)	1,753.5 (384–3,765.25)	0.105
	max	1,901 (548.2–032.75)	1,342 (428.75–6,765.2)	3,728.5 (816.2–5,565.2)	0.328
	d/c	103 (49.8–223.5)	110.5 (61.4–267.25)	63.8 (32–153)	0.092
Albumin (mg/dL)	adm	36.75 (32–42.55)	37.79 (32.6–43.13)	34.5 (32–40.08)	0.192
	min	30 (24–34.83)	31 (23.68–35.22)	27.75 (24.48–32.48)	0.413
	d/c	38.54 (35.08–40.14)	38.5 (34.62–40.05)	38.9 (36.05–40.76)	0.606
Sodium (mmol/L)	adm	134.4 ± 4.9 (120–146)	134.6 ± 5.3 (120–146)	133.8 ± 4 (128–143)	0.480*
	min	132 (130–135)	132 (130–135)	133 (130–135)	0.669
	d/c	137 (136–138)	137 (135.75–138)	137 (136–138)	0.818
LDH (IU/L)	adm	278 (230.5–315.5)	270.5 (224.75–305)	294 (244.25–378.25)	0.056
	max	314.5 (275.5–393.5)	301 (264.75–361.75)	345.5 (294.75–438)	**0**.**009**
	d/c	235.5 ± 60.76 (117–435)	235 ± 55.99 (126–435)	236.6 ± 71.05 (117–404)	0.906*
ALT (IU/L)	adm	17 (11.75–34)	16 (11–29)	28.5 (12–57.75)	**0**.**048**
	d/c	24 (14–44.75)	26 (14.75–49.75)	21.5 (13.25–36.25)	0.190
AST (IU/L)	adm	26 (17.75–38.25)	24.5 (17.75–34.25)	32 (17.75–57.75)	0.075
	d/c	22 (16–28.25)	22 (17.75–29.5)	21.5 (14.25–26.75)	0.333
Urea (mg/dL)	adm	21.5 (16–19)	22.5 (16–30)	20.5 (15.25–27.75)	0.564
	d/c	27.85 ± 9.45 (6–58)	27.49 ± 9.58 (6–58)	28.66 ± 9.24 (11 53)	0.477*
Creatinine (mg/dL)	adm	0.51 (0.4–0.68)	0.51 (0.44–0.66)	0.57 (0.37–0.71)	0.834
	d/c	0.42 (0.35–0.5)	0.43 (0.35–0.5)	0.41 (0.35–0.51)	0.697
Amylase (U/L)^b^	adm	41 (31–57)	44 (32- 60)	38 (27–52.75)	0.191
	max	74 (53–118.5)	71 (49–109.5)	75 (56.25–142.25)	0.228
	d/c	68.5 (49.5–103.5)	70 (50.5–103)	66 (47–112)	0.971
Lipase (U/L)^b^	adm	18.3 (12.6–31.9)	18 (12.5–30.55)	18.65 (12.88–37.1)	0.530
	max	35.6 (25.25–78.55)	32.7 (23.35–68.5)	54.85 (26.1–140.13)	0.05
	d/c	31.5 (24.75–58)	31 (24.5–53.4)	32.5 (23–71)	0.585
D-Dimer (ng/mL)	adm	919 (512.2- 1,670.5)	875 (429.25–1,577.5)	199.5 (611–1,798.75)	0.091
	max	1,460 (771.75–2,394)	1,209.5 (696.75–2,167)	1,897 (1,033–2,948.2)	**0**.**027**
	d/c	214 (136.5–358.5)	216 (141.25–340.25)	214 (124–374)	0.831

CRP, C-reactive protein; PCT, procalcitonin; ESR, erythrocyte sedimentation rate; IL-6, interleukin-6; BNP, B-type natriuretic peptide; LDH, lactate dehydrogenase; AST, aspartate aminotransferase; ALT, alanine aminotransferase adm: at the time of admission, **d/c**: at the time of discharge; max, maximum; min, minimum.

The *p*-values in bold are statistically significant.

^a^ Total number of patients:58, first wave cohort:28, second wave cohort:30.

^b^ Total number of patients:70, first wave cohort:45, second wave cohort:25.

* The *p*-value was obtained from the Student's *t*-test and results are given as mean ± SD (min-max). Other *p*-values were obtained from the Mann–Whitney *U*-test. Other results are given as median (IQR).

**Table 3:** Frequency of pathological laboratory findings in patients.

Variables	Study group *n* (%)		First wave cohort *n* (%)	Second wave cohort *n* (%)	*p*-value
Elevated PCT	adm	80 (%78.4)	53 (%75.7)	27 (%84.4)	0.324
	max	88 (%86.3)	61 (%87.1)	27 (%84.4)	0.760
Elevated ESR	adm	41 (%40.2)	20 (%28.6)	21 (%65.6)	**<0**.**001**
Elevated IL-6^a^	adm	63 (%79.7)	36 (%73.5)	27 (%90)	0.076
	max	66 (%80)	38 (%84.4)	28 (%93.3)	0.301
Elevated Ferritin	adm	70 (%68.6)	45 (%64.3)	25 (%78.1)	0.162
	max	82 (%80.4)	55 (%78.6)	27 (%84.4)	0.493
Anemia	adm	14 (%13.7)	9 (%12.9)	5 (%15.6)	0.760
Leukocytosis	adm	26 (%25.5)	21 (%30)	5 (%15.6)	0.122
Lymphopenia	adm	48 (%47.1)	32 (%45.7)	16 (%50)	0.687
	min	72 (%70.6)	50 (%71.4)	22 (%68.8)	0.783
Thrombocytopenia	adm	25 (%24.5)	12 (%17.1)	13 (%40.6)	**0**.**011**
	min	38 (%37.3)	21 (%30)	17 (%53.1)	**0**.**025**
Elevated Troponin	adm	32 (%31.4)	22 (%31.4)	10 (%31.3)	0.986
	max	53 (%52)	38 (%54.3)	15 (%46.9)	0.487
Elevated BNP	adm	47 (%46.1)	28 (%40)	19 (%59.4)	0.069
	max	75 (%73.5)	48 (%68.6)	27 (%84.4)	0.093
Hypoalbuminemia	adm	20 (%19.6)	14 (%20)	6 (%18.8)	0.883
	min	72 (%70.6)	46 (%65.7)	26 (%81.3)	0.110
Hyponatremia	adm	51 (%50)	32 (%45.7)	19 (%59.4)	0.200
	min	58 (%56.9)	41 (%58.6)	17 (%53.1)	0.606
Elevated LDH	adm	58 (%56.9)	35 (%50)	23 (%71.3)	**0**.**038**
	max	82 (%80.4)	53 (%75.7)	29 (%90.6)	0.078
Elevated transaminase	adm	17 (%16.7)	7 (%10)	10 (%31.3)	**0**.**008**
Elevated renal function tests	adm	11 (%10.8)	8 (%11.4)	3 (%9.4)	0.527
Elevated amylase and/or lipase^b^	adm	36 (%40.4)	21 (%36.8)	15 (%46.9)	0.355
Elevated D-Dimer	adm	76 (%74.5)	50 (%71.4)	26 (%81.3)	0.291
	max	77 (%75.5)	51 (%72.9)	26 (%81.3)	0.361
Proteinuria and/or hematuria	adm	4 (%3.9)	4 (%5.7)	0 (%0)	0.306

CRP, C-reactive protein; PCT, procalcitonin; ESR, erythrocyte sedimentation rate; IL-6, interleukin-6; BNP, B-type natriuretic peptide; LDH, lactate dehydrogenase, adm: at admission hospital; max, maximum; min, minimum.

The *p*-values in bold are statistically significant.

^a^ Total number of patients:58, first wave cohort:28, second wave cohort:30.

^b^ Total number of patients:70, first wave cohort:45, second wave cohort:25.

Polymerase chain reaction (PCR) testing and serology for SARS-CoV-2 were positive in two (2.9%) and 63 (90%) patients in the first-wave cohort; while four (12.5%) and 29 (90.6%) patients were positive in the second-wave cohort, respectively (*p* = 0.076, *p* = 1).

The ECG and echocardiography findings of the patients at the time of admission are given in Table 4. There was no significant difference between the first-wave and second-wave cohorts in ECG pathologies (*p* > 0.05). Mitral valve regurgitation (*p* = 0.394) and coronary artery involvement (*p* = 0.394) were more common in the first-wave cohort, whereas left ventricular dysfunction (*p* = 0.462) and pericardial effusion were more common in the second-wave cohort (*p* = 0.017) (Table 4). There was no significant difference between the cohorts in chest radiography and abdominal ultrasonography findings (Table 4).

**Table 4:** Electrocardiography and imaging findings of the patients.

Variables	Study group *n* (%)	First wave cohort *n* (%)	Second wave cohort *n* (%)	*p*-value
Pathology findings in electrocardiography	53 (%52)	35 (%50)	18 (%56.3)	0.558
Sinus tachycardia	33 (%32.4)	22 (%31.4)	11 (%34.4)	0.768
1st-degree block	24 (%23.5)	18 (%25.7)	6 (%18.8)	0.442
Sinus bradycardia	15 (%14.7)	10 (%14.3)	5 (%15.6)	0.538
ST-T changes	12 (%11.8)	7 (%10)	5 (%15.6)	0.413
QT prolongation	10 (%9.8)	7 (%10)	3 (%9.4)	0.922
Pathology findings in echocardiography	68 (%66.7)	43 (%61.4)	25 (%78.1)	0.097
Mitral regurgitation	28 (%27.5)	21 (%30)	7(%21.9)	0.394
Coronary artery lesion	19 (%18.6)	16 (%22.9)	3 (%9.4)	0.105
Aneurysm	7 (%6.9)	6 (%8.6)	1 (%3.1)	0.231
Dilatation	12 (%11.8)	10 (%14.3)	2 (%6.2)	
Systolic dysfunction	36 (%29.4)	23 (%27.1)	13 (%34.4)	0.462
Left ventricular ejection fraction median (IQR)	63 (58–67)	63 (58–67.25)	62.5 (58.25–65.75)	0.433*
Pericardial effusion	40 (%39.2)	22 (%31.4)	18 (%56.3)	**0**.**017**
Pathology findings in posteroanterior (PA) chest radiography	15 (%14.7)	11 (%15.7)	4 (%12.5)	0.667
Infiltration	8 (%7.8)	6 (%8.6)	2 (%6.2)	0.847
Pulmonary edema	5 (%4.9)	4 (%5.7)	1 (%3.1)	
Pleural effusion	2 (%2)	1 (%1.4)	1 (%3.1)	
Pathology findings in abdominal ultrasonography	29/48 (%56.9)	17/23 (%65.4)	12/25 (%48)	0.210
Intra-abdominal fluid	12 (%25)	10 (%43.5)	2 (%8)	0.065
Lymphadenopathy	11(%22.9)	4 (%17.4)	7 (%28)	
Cholecystitis	3 (%6.2)	2 (%8.7)	1 (%4)	
Renal hyperechogenicity	1 (%2.1)	1 (%4.3)	0 (%0)	
Appendicitis	1 (%2.1)	0 (%0)	1 (%4)	

The *p*-values in bold are statistically significant.

* *p*-value was obtained from the Mann–Whitney *U*-test.

At the time of diagnosis, four (3.9%) patients, two each in the first and second-wave cohorts, met the criteria for complete KD. Furthermore 12 (17.1%) patients in the first-wave cohort and four (12.5%) patients in the second-wave cohort, met the criteria for incomplete KD (*p* = 0.082). SARS-CoV2 serology was positive in all complete KD patients. Among the incomplete KD patients, SARS-CoV2 PCR was positive in only one patient, and SARS-CoV2 serology was positive in the other patients.

Our patients were followed up in the hospital for a median duration of 10 (8–13) (min.3-max.30) days. A total of 27 (26.5%) patients, 19 (27.1%) in the first-wave cohort and 8 (25%) in the second-wave cohort, were followed up in the ICU (*p* = 0.82). Median LoS was 10 (7–11, 13) (min.4-max.28) days in the first-wave cohort and 10.5 (8–14) (min.3-max.30) days in the second-wave cohort (*p* = 0.121). None of the patients needed mechanical ventilation or extracorporeal membrane oxygenation, and none had died.

As an immunomodulatory treatment, a total of 50 (49%) patients, 36 (51.4%) in the first-wave cohort and 14 (43.7%) in the second-wave cohort, were given steroid treatment in combination with intravenous immunoglobulin (IVIG). On the other hand, a total of 26 (25.5%) patients, 17 (24.3%) in the first-wave cohort and nine (28.1%) in the second-wave cohort, were given only steroids, and a total of 6 (5.8%) patients, three (4.3%) in the first-wave cohort and three (9.4%) in the second-wave cohort, were given only IVIG treatment. A total of 11 (10.8%) patients, eight (11.4%) in the first-wave cohort and three (9.4%) in the second-wave cohort, were given Anakinra. No immunomodulatory treatment was given to a total of nine (8.8%) patients with mild clinical findings. There was no significant difference between the cohorts in terms of immunomodulatory treatment (*p* = 0.845). Inotropic therapy was given to 20 (28.6%) patients in the first-wave cohort and nine (29%) in the second-wave cohort whose hypotension did not respond to fluid therapy (*p* = 0.962). The median vasoactive-inotropic score was 20 (11–25) in the first-wave cohort, it was 17 (7–22.5) in the second-wave cohort (*p* = 0.254). Inotropic therapy was given to median 3.5 (3–5) days patients in the first-wave cohort and 3 (2–5) days in the second-wave cohort (*p* = 0.326). These parameters were found to be similar in both attacks.

Clinical findings improved, and laboratory parameters regressed in all patients at discharge. There was no significant difference between the cohorts in laboratory parameters measured at discharge (Table 2). Echocardiographic evaluation at discharge revealed pathology in 20 (28.6%) and 10 (31.3%) patients in the first and second-wave cohorts, respectively (*p* = 0.788). Left ventricular systolic functions were normal in all patients. Minimal pericardial effusion was still present in 10 (14.3%) and nine (29.4%) patients in the first and second-wave cohorts, respectively (*p* = 0.096). Mild mitral valve regurgitation was present in eight (11.4%) and only one (3.1%) patient in the first and second-wave cohorts, respectively (*p* = 0.266). In addition, compared to only one (3.1%) patient who had a coronary artery aneurysm in the second-wave cohort, six (8.6%) patients had coronary artery aneurysm, and four (5.7%) patients had coronary artery dilatation in the first-wave (*p* = 0.110).

Echocardiographic evaluation performed at the sixth-month follow-up visit revealed a coronary artery aneurysm in one (3.1%) patient in the second-wave cohort. Additionally, it was determined that three (4.3%) had coronary artery dilatation, and one (1.4%) had coronary artery aneurysm in the first-wave cohort (*p* = 0.276). At the twelfth-month evaluation, it was observed that coronary aneurysm persisted in one patient each in the first and second-wave cohorts (*p* = 0.538). One of the two patients whose coronary aneurysm persisted at 6 and 12 months met the criteria for complete KD, while the other did not meet the criteria for KD. While two of our patients with coronary dilatation met the criteria for incomplete KD, the other did not meet the criteria for KD.

## Discussion

One hundred two patients, who were diagnosed with MIS-C in a single center, were divided into two groups: the first MIS-C wave cohort and the second MIS-C wave cohort. The demographic, clinical, and laboratory findings of these cohorts obtained at admission, discharge, and follow-up were compared. The clinical findings have not differed significantly between the cohorts, except for respiratory system involvement, which was more frequent in the first-wave cohort. However, significant variations in laboratory findings were noted, with the second-wave cohort exhibiting significantly higher levels of IL-6, ESR, LDH, ALT, and D-Dimer. Thrombocytopenia and pericardial effusion were significantly more frequent in the second-wave cohort than in the first-wave cohort. On the other hand, the cohorts have not differed significantly in terms of MIS-C treatments, LoS, the need for intensive care, and outpatient follow-up results.

It has been speculated that the different variants of the SARS-CoV-2 virus circulating during the time of diagnosis of MIS-C patients may affect the MIS-C clinic (5, 6, 13). In this context, Jain et al. compared the MIS-C patients affected by original/alpha, delta variants of the SARS-CoV-2 virus and observed that the musculoskeletal and respiratory system symptoms, as well as the findings like in KD, were more frequent in the wave featuring the original/alpha variant. Additionally, patients diagnosed during the delta variant wave had a significantly shorter ICU stay (5). In a prospective study conducted in Denmark, the clinical features of MIS-C patients were compared between the cohorts diagnosed during the waves featuring the alpha and delta variants (6). Consequentially, it was found that the respiratory system involvement was less in MIS-C patients diagnosed during the wave featuring the delta variant; however, the difference between the cohorts was not significant. It was also reported in the same study that the cohorts have not differed in other clinical findings, except for LoS, which was shorter in MIS-C patients diagnosed during the wave featuring the delta variant (6). In comparison, the COVID-19 infection data collected for this study were not categorized according to the variants of the SARS-CoV-2 virus. Although variant data were not available for our cohort, the lower frequency of respiratory involvement in the second-wave may possibly be related to the predominance of the Delta variant during that period. The delta variant, which was officially started to be detected in Turkey as of April 2021, as compared to the alpha variant, which was officially started to be detected in Turkey as of January 2021 (14).

In a study conducted with 106 patients hospitalized with the diagnosis of MIS-C, Harahsheh et al. found that patients diagnosed during the second-wave presented to the hospital with higher maximum troponin and N-terminal BNP values and more frequently needed advanced respiratory and inotropic support (4). They argued that patients diagnosed during the second-wave experienced potentially intermittent or multiple repeated exposures to the circulating SARS-CoV-2 virus and that this might have led to repeated stimulation of the immune system (4). We think that the finding of elevated levels of acute phase reactants (ESR, IL-6) in the second-wave cohort included in this study supports the hypothesis. Repeated exposures may stimulate the dysregulated hyperimmune response characteristic of MIS-C.

In a study conducted with 4,470 MIS-C patients in the United States, it was reported that the severity of the disease decreased in time over the course of three COVID-19 waves (1). The same study reported an increase in the number of patients with hematologic and gastrointestinal involvement, along with a temporal decline in cardiac dysfunction, myocarditis, vasopressor use, and the length of stay in both the hospital and the intensive care unit (1). It has been suggested that the variations observed between waves may be attributable to the progressive refinement of disease definition and treatment strategies over time. Differences in findings may also stem from changes in diagnostic testing methods, and the recognition of new clinical manifestations may have further contributed to these variations (1, 5). Moreover, the improvement in clinical outcomes has been associated with the identification of milder MIS-C cases over time, heightened awareness of the disease facilitating earlier diagnosis, and advancements in the clinical management of MIS-C (1, 4). In comparison, a standard protocol was followed in identifying and treating MIS-C patients since the first MIS-C patient was identified in the clinic where this study was conducted. It might be that the standardized protocol and the definition of the disease played a role in the fact that the hospitalization, treatment and follow-up results were similar despite the elevation in the laboratory parameters of the second-wave cohort. In addition, the fact that the study sample also included the patients with mild involvement since the first MIS-C patient might have also played a role in the fact that the clinical findings were similar.

Harahsheh et al. also reported similar coronary artery abnormalities and systolic dysfunction in MIS-C patients diagnosed during both waves (4). On the other hand, Miller et al. reported that systolic dysfunction decreased over time, but there was no significant difference between the cohorts pertaining to different waves in terms of pericardial effusion and coronary artery abnormalities (1). In comparison, the echocardiographic findings of the patients included in this study were evaluated in detail. Accordingly, mitral valve regurgitation and coronary artery involvement were more frequent in the first-wave cohort, whereas the left ventricular dysfunction was more frequent in the second-wave cohort. However, the said differences between the cohorts were not significant. On the other hand, pericardial effusion/pericarditis was significantly more common in the second-wave cohort. Higher frequencies of both systolic dysfunction and pericardial effusion/pericarditis in the second-wave cohort may be an indication of the hyperimmune response triggered by repeated exposure.

The mid-long-term results of MIS-C patients have begun to be reported in the literature, yet there is still no study that compared these results between the cohorts pertaining to different waves (15–17). In a study featuring a 4- to 9-month follow-up period, mild ventricular dysfunction and mild atrioventricular valve insufficiency were found in one patient each, and no dilatation or aneurysm was observed in the coronary arteries (15). In another study featuring a sixth-month follow-up, it was found that coronary artery abnormalities persisted in three patients (16). Yet, in another study, a coronary artery aneurysm and a pericardial effusion were detected in two patients at the sixth-month follow-up assessment (17). Hence, this study is the first to compare mid-long term outcomes between the cohorts pertaining to different waves. The echocardiographic findings of the patients have not differed significantly between the outpatient follow-ups.

The most important limitation of this study was the lack of data on variants of the SARS-CoV-2 virus. Other limitations are that the evaluation of peripheral T cell subtypes was not conducted to support the diagnosis of MIS-C and that it was as a single-center, retrospective study.

## Conclusions

The findings of this study revealed a significant increase in laboratory parameters of the MIS-C patients in time throughout the COVID-19 waves. However, there was no significant difference between the cohorts about pertaining to different waves in terms of clinical findings and treatments. The echocardiographic findings of the patients have also not differed significantly between the outpatient follow-ups.

## Data Availability

The raw data supporting the conclusions of this article will be made available by the authors, without undue reservation.
